# Artificial intelligence against the first wave of COVID-19: evidence from China

**DOI:** 10.1186/s12913-022-08146-4

**Published:** 2022-06-10

**Authors:** Ting Wang, Yi Zhang, Chun Liu, Zhongliang Zhou

**Affiliations:** 1 Jinhe Center for Economic Research, Xi’an Jiaotong University, No. 28 Xianning West Road, Xi’an, Shaanxi 710049 People’s Republic of China; 2 School of Economics, Southwestern University of Finance and Economics, No. 555 Liutai Avenue, Wenjiang District, Chengdu, Sichuan 611130 People’s Republic of China; 3 School of Public Policy and Administration, Xi’an Jiaotong University, No. 28 Xianning West Road, Xi’an, Shaanxi, 710049 People’s Republic of China

**Keywords:** Artificial intelligence, COVID-19, Prevention, China

## Abstract

**Background:**

The COVID-19 pandemic unexpectedly broke out at the end of 2019. Due to the highly contagious, widespread, and risky nature of this disease, the pandemic prevention and control has been a tremendous challenge worldwide. One potentially powerful tool against the COVID-19 pandemic is artificial intelligence (AI). This study systematically assessed the effectiveness of AI in infection prevention and control during the first wave of COVID-19 in China.

**Methods:**

To better evaluate the role of AI in a pandemic emergency, we focused on the first-wave COVID-19 in the period from the early December 2019 to the end of April 2020 across 304 cities in China. We employed three sets of dependent variables to capture various dimensions of the effect of AI: (1) the time to the peak of cumulative confirmed cases, (2) the case fatality rate and whether there were severe cases, and (3) the number of local policies for work and production resumption and the time span to having the first such policy. The main explanatory variable was the local AI development measured by the number of AI patents. To fit the features of different dependent variables, we employed a variety of estimation methods, including the OLS, Tobit, Probit, and Poisson estimations. We included a large set of control variables and added interaction terms to test the mechanisms through which AI took an effect.

**Results:**

Our results showed that AI had highly significant effects on (1) screening and detecting the disease, and (2) monitoring and evaluating the epidemic evolution. Specifically, AI was useful to screen and detect the COVID-19 in cities with high cross-city mobility. Also, AI played an important role for production resumption in cities with high risk to reopen. However, there was limited evidence supporting the effectiveness of AI in the diagnosis and treatment of the disease.

**Conclusions:**

These results suggested that AI can play an important role against the pandemic.

**Supplementary Information:**

The online version contains supplementary material available at 10.1186/s12913-022-08146-4.

## Background

In December 2019, the Corona Virus Disease 2019 (COVID-19), caused by severe acute respiratory syndrome corona virus 2 (SARS-CoV-2), broke out and spread rapidly in Wuhan, China. Globally more than 474.660 million people have been confirmed with the disease and 6.103 million died in this disaster as of March 24, 2022 [1]. Despite the positive news about COVID-19 vaccines utilized for mass inoculations across the world, they are not yet a silver bullet because of the vaccine inequality, the vaccine hesitancy, and the virus mutations [2–6]. The influence of the pandemic, a once-in-a-century health crisis, will last for a long time and disaster response efforts around the world are urgently needed [7]. This raises the important question of how to effectively prevent and control the spread and persistence of highly contagious epidemic diseases like COVID-19. Finding powerful instruments in the pandemic emergency, therefore, ranks high on any health care agenda.

**Fig. 1:**
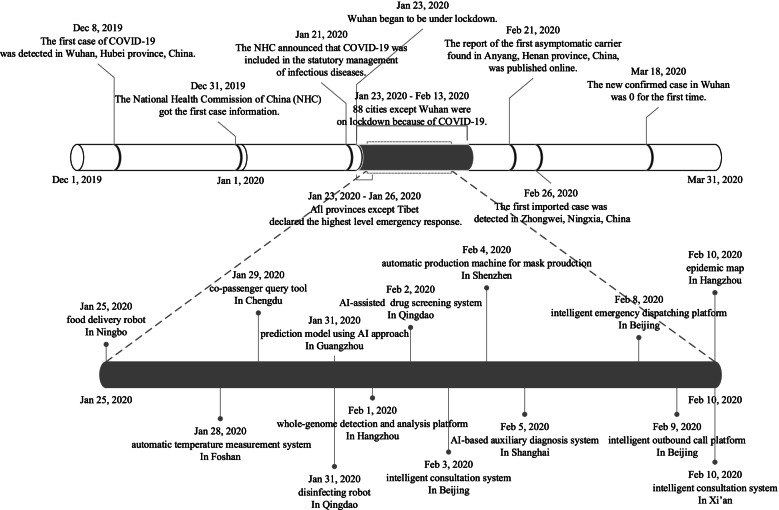
Timeline of COVID-19 and relevant AI applications. Source: Online news collection

World Health Organization (WHO) has acknowledged the efforts made by the Chinese government to investigate and contain the pandemic [8]. Besides the lock-down of cities and the built-up of specialized hospitals, one instrument employed widely in China to fight against COVID-19 is artificial intelligence (AI). Figure 1 illustrated some of the AI applications in China during the first wave of the pandemic (from early December 2019 to the end of April 2020).

The applications can be summarized in three ways. First, AI can facilitate screening and detecting the pandemic. For example, data mining technology has been used to track individual mobility by gathering information on consumption and travel histories [9, 10]. Intelligent outbound call platforms, based on intelligent speech semantic technologies, can automatically collect key information, such as residential activity areas, contact groups, and whether typical symptoms occur, and conduct statistical analysis via man–machine interaction. Also, automatic body temperature measurement devices using image recognition and infrared thermal imaging technology help to perform real-time non-contact detection to quickly identify and screen people with abnormal body temperature.

Second, the application of AI in diagnosis and treatment may improve medical efficiency [11, 12]. After using the image recognition technique in the automatic detection of the diseased area, the analysis time can be shortened from 5–6 h to 2–3 s [13], which improves the efficiency of accurate quantitative analysis and reduces the risk of hospital cross-infection [14]. To speed up and improve the accuracy of the nucleic acid test, the AI-driven whole-genome detection technology has established a protein three-dimensional structure prediction model for virus genes, like LinearFold and BiLSTM + DNN model [14–16], to perform whole-genome sequence analysis and sample comparison. In addition, the AI-assisted drug screening system can be used to conduct computational screening of listed drugs and obtain potentially effective drugs with better scores [12, 14, 17, 18].

Third, AI can be used to monitor and evaluate the epidemic evolution, which is crucial for work and production resumption plans [19, 20]. For instance, an intelligent data visualization platform, i.e. the epidemic map [21, 22], is indispensable for efficiently and effectively classifying the risk of infection across regions. The intelligent emergency dispatching platform facilitates the distribution of materials by collecting statistics and monitoring the logistics system. Meanwhile, the application of intelligent road sweepers, drone transportation, and intelligent patrol robots greatly contributes to the epidemic prevention and control after the work and production resumption.

Despite the important role of AI in pandemic emergency management, relatively limited evidence on this topic has been provided. This study aimed to narrow this research gap by investigating the effects of AI on COVID-19 prevention and control. We contribute to the existing literature in two aspects. First, to the best of our knowledge, this is one of the few studies to empirically examine the effect of AI in epidemic prevention and control. By controlling for a large set of covariates and applying a variety of estimation methods, including the ordinary least squares (OLS), Tobit, Probit, and Poisson regressions, we found city-level evidence from China supporting the positive and significant impact of AI on the prevention and control of the first-wave COVID-19. Second, using interaction terms between AI and several mechanism variables, we explored the channels through which AI played a role. The results showed that AI not only helped to screen and detect the COVID-19, but also facilitated the monitoring and evaluation of the epidemic evolution. We proposed that our findings offered new insights into the role of digital technologies and especially AI in dealing with major clinical problems and diseases. Moreover, this study also provided important policy implications for government disaster response and healthcare emergency management.

## Methods

This study focused on 304 cities from 31 provinces in China. We excluded the city of Wuhan because it was the center of the epidemic in China and therefore showed a very different pattern in pandemic prevention and control compared with other cities. Table 1 provided the description and sources of the main variables.

**Table 1:** Variable definitions

Variable	Definition
*Dependent variables*	
TTP	The time to the peak of the cumulative confirmed cases for each city excluding asymptomatic carriers and imported cases from abroad. *Source*: 31 provincial Health Commissions in China [23]
CFR (%)	The proportion of people who died from COVID-19 among all individuals diagnosed with the disease excluding asymptomatic carriers and imported cases from abroad till the end of April 2020. *Source*: 31 provincial Health Commissions in China [23]
Severe cases	A dummy variable that equals 1 if the city had severe cases excluding asymptomatic carriers and imported cases from abroad till the end of April 2020, 0 otherwise. *Source*: 31 provincial Health Commissions in China [23]
Number of policies	The cumulative number of policies on production resumption in each city till 31 March 2020 (The time was selected according to China Urban Vitality Research Report, 2020Q1) *Source*: COVID-19 Graph-Knowledge Dashboard [24]
Time span	The time span from the last day of the Spring Festival to the day when the local government introduced the first policy on production resumption. (The negative value represented the first policy on production resumption had been introduced before the end of the holiday. In order to facilitate the empirical analysis, we rescaled this variable and made the values positive.) *Source*: COVID-19 Graph-Knowledge Dashboard [24]
*Independent variable*	
AI	The logarithm of the local AI development. The level of AI development was measured by the cumulative number of AI-related patents applied from 2012 to 2019 at the provincial level. (AI patents were authorized patents related to artificial intelligence.) *Source*: Patsnap [25]
*Mechanism variables*	
Migration	Human migration rate which was measured by the Baidu Migration index to represent the total intensity of migration from Wuhan to other cities before Wuhan was locked down (from 1 January 2020 to 24 January 2020) *Source*: Baidu Migration [26]
Public transit volume	The logarithm of the volume of local public transportation in the municipal district of each city. *Source*: China City Statistical Yearbook [27]
Number of COVID-19 hospitals	The number of hospitals for COVID-19 in the municipal district of each city (per 10,000 people). *Source*: CSMAR [28]
Number of firms	The logarithm of the number of industrial enterprises above designated size in the municipal district of each city. *Source*: China City Statistical Yearbook [27]
*Control variables*	
*Macro characteristics*	
GDP per capita	The logarithm of GDP per capita at the city level. *Source*: China City Statistical Yearbook [27]
Population density	The population density in the municipal district of each city (10,000 people per square kilometer). *Source*: China City Statistical Yearbook [27]
*Health care resources*	
Proportion of public employment	The density of employment in public sectors (per 10,000 people). *Source*: China City Statistical Yearbook [27]
Number of Three-A hospitals	The number of Three-A hospitals in the municipal district of each city (per 10,000 people). *Source*: 31 provincial Health Commissions in China [23]
*Epidemic severity*	
Lockdown	A dummy variable that equals 1 if the local government or various media news in 2020 provided lockdown information for this city, 0 otherwise. *Source*: News media and government announcements [29]
Confirmed cases	The logarithm of the number of confirmed cases excluding asymptomatic carriers and imported cases from abroad till the end of April 2020. *Source*: 31 provincial Health Commissions in China [23]

## Measures

### Dependent variables

We employed three sets of dependent variables to capture the role of AI in COVID-19 prevention and control.

1. Time to the peak of the cumulative confirmed cases (*TTP*).
  According to Adda (2016) [30], effective prevention and control of an epidemic can largely shorten the time to reach the peak of the cumulative confirmed cases. Therefore, the earlier arrival of the peak time can be regarded as an indicator of the effective prevention and control of COVID-19. We calculated the time to the peak of the cumulative confirmed cases for each city and denoted this variable as *TTP*. Data on this variable was manually collected from the Health Commission in each province. We excluded asymptomatic carrier cases and imported cases from abroad in calculation.
2. Case fatality rate (*CFR*) and whether there were severe cases (*Severe cases*).
  Mortality and severity are two of the most important indicators in epidemiology, both can be influenced by early diagnosis and control [30–32]. We proposed that lower levels of mortality and severity would suggest effective efforts in disease control and prevention. We calculated the case fatality rate (*CFR*) and employed a dummy variable (*Severe cases*) to denote whether there were severe cases. We took the data from the Health Commission in each province covering the period from the early December 2019 to the end of April 2020 (the first wave of COVID-19 in mainland China). Also, we excluded asymptomatic carrier cases and imported cases from abroad in calculation.
3. Number of local policies on production resumption (*Number of policies*) and the time span to having such policies (*Time span*).
  Stringent quarantine at the cost of economic stagnation is not a long-term solution, particularly for developed regions. Production resumption depends not only on the effectiveness of the epidemic prevention and control, but also on the local capability to respond to the potential rebound risk of the epidemic after the resumption of work [32, 33]. It is plausible that a larger number of local policies on production resumption and a shorter time span to having such policies reflect better prevention and control of COVID-19. We constructed two variables, the number of local policies on production resumption (*Number of policies*) and the time span to having such policies (*Time span*), respectively. Data was taken from COVID-19 Graph-Knowledge Dashboard, a knowledge-based global COVID-19 epidemic risk prediction and work resumption decision-making system created by the AMiner Team in the Department of Computer Science, Tsinghua University.

### Independent variables

We used the cumulative number of patents on AI technologies (*AI*) applied from 2012 to 2019 at the provincial level to measure the development of artificial intelligence [34, 35]. The data was obtained from PatSnap, a global patent platform with a daily update covering 116 countries from 1867. This database has been used in many recent studies on innovation [36, 37]. According to *China AI Development Report 2018*, patents on AI in China began to rise rapidly since 2012. Therefore, we chose 2012 as the start year. In order to alleviate the endogeneity issue due to reverse causality, we used AI patents up to 2019, the year before the outbreak of the COVID-19. To identify how AI helped to prevent and control the COVID-19, we generated three sets of interaction variables between AI and mechanism variables.

1. AI interacted with cross-border mobility (*AI* × *Migration*) and with within-city mobility (*AI* × *Public transit volume*)
  The infection of COVID-19 was of clustering onset and most cases were reported to have Wuhan-related exposures in the first wave [38–40]. This suggested that efficient screening and early detection of COVID-19 were crucial to prevent onward transmission. We proposed that effective epidemic screening and detecting would be more important in cities with higher levels of cross-border and/or within-city mobility. In particular, we constructed two variables to measure different patterns of mobility. First, following Qiu et al. (2020) [41], we took information from Baidu Migration on the total intensity of migration from Wuhan to other cities before Wuhan was locked down to measure the cross-border mobility (*Migration*). We set January 1, 2020 as the starting point and January 24, 2020 (i.e., the date of lockdown in Wuhan) as the end point of our study window to account for the potential transmission in the early period with no intervention in place. Second, due to the lack of information on within-city mobility during the epidemic period, we used the most recent data on the volume of local public transportation from the China City Statistical Yearbook to proxy for the intensity of within-city mobility (*Public transit volume*). We then generated two interaction terms between AI and these two mobility variables. We used the time to the peak of the cumulative confirmed cases (*TTP*) as the dependent variable in this test. Because effective contact tracing and case isolation are more important in cities with higher levels of mobility, AI could help to shorten the time to the peak of the cumulative confirmed cases especially in these cities. We therefore would expect to observe significantly negative coefficients on the interaction of AI and cross-border mobility (*AI*×*Migration*) and that of AI and within-city mobility (*AI*×*Public transit volume*).
2. AI interacted with the number of COVID-19 hospitals (*AI* × *Number of COVID-19 hospitals*)
  Early diagnosis and timely treatment are crucial for reducing the risk of COVID-19. Nevertheless, the effectiveness of diagnosis and treatment depends on the availability of local health care resources, especially those pertinent to COVID-19. We took the data on the number of hospitals for treating COVID-19 from the Chinese Stock Market Research (CSMAR) database to measure the availability of COVID-19 related health care resources (*Number of COVID-19 hospitals*). We then constructed an interaction between AI and this variable (*AI*×*Number of COVID-19 hospitals*). Both the case fatality rate (*CFR*) and whether there were severe cases (*Severe cases*) were used as the dependent variables. As cities with more health care resources could provide better diagnosis and treatment, we would expect this interaction to be significantly positive if improving diagnosis and treatment of COVID-19 was an important channel through which AI played a role. In other words, in cities with higher availability of COVID-19 related health care resources, AI might have a stronger effect on diagnosing the disease and monitoring the treatment, which could lower the case fatality rate and the probability of having severe cases.
3. AI interacted with the number of local firms (*AI* × *Number of firms*)
  The potential rebound risk of the epidemic would make the local government more hesitant in work resumption. Hence, cities with higher demand or larger risk for reopening are more contingent on better monitoring and evaluation of the disease. In these cities, with the help of AI, local government officials would be more confident to reopen the economy with a larger number of policies on work resumption and a shorter relative time span to having such policy effort. We therefore proposed that AI played a larger role in monitoring and evaluating the epidemic situation in cities with higher demand or risk for reopening. We used the number of local industrial firms in a city to measure the potential demand (and also the risk) for work resumption (*Number of firms*). Data on this variable was obtained from the most recent China City Statistical Yearbook. We then generated an interaction between this variable and AI (*AI* × *Number of firms*). We would expect this interaction to be significantly positive when the number of local policies on production resumption (*Number of policies*) was used as the dependent variable, and negative when we used the time span to having such policies (*Time span*) as the dependent variable.

### Control variables

To alleviate the omitted variables bias in our regressions, we controlled for a large set of city characteristics. First, we added two variables on local macro situation including city-level gross domestic product per capita (*GDP per capita*) and population density (*Population density*) [30, 41]. We expected that it was more difficult to prevent and control the epidemic in more developed and populous cities. Following extant research linking general health care resources to epidemic controls [42], we then controlled for the proportion of the employment in public sectors (*Proportion of public employment*) and the number of Three-A hospitals per 10,000 people (*Number of Three-A hospitals*). In addition, we added two variables on the epidemic severity in each city including whether the city was once locked down in 2020 (*Lockdown*) and the number of confirmed cases (*Confirmed cases*) [43, 44].

### Estimation strategy

To investigate the effect of AI on COVID-19 prevention and control, we set up the following econometric model:

1 $$Y_{i} = \beta_{0} + \beta_{1} AI_{p} \times mechanism_{i} + \beta_{2} mechanism_{i} + \Phi Controls_{i} + \sigma_{p} + \varepsilon_{i}$$

where *i* and *p* denoted city and province, respectively. In our model specification, *Y*_*i*_ represented for dependent variables including the time to the peak of the cumulative confirmed cases (*TTP*), the case fatality rate (*CFR*), whether there were severe cases (*Severe cases*), the number of local policies on production resumption (*Number of policies*), and the time span to having such policies (*Time span*). *AI*_*p*_ was the key explanatory variable measuring the provincial level of AI development. Our mechanism variables (*mechanism*_*i*_) included the importance of screening and detecting the epidemic (*Migration*; *Public transit volume*), the availability of local COVID-19 related health care resources (*Number of COVID-19 hospitals*), and the local demand for work resumption (*Number of firms*). *Controls*_*i*_ denoted for a set of variables on local macro characteristics, general health care resources, and epidemic severity. In addition, we controlled for the province fixed effects (*σ*_*p*_). *ɛ*_*i*_ was the random error term. As explained in the previous subsection, the variable of interest in Eq. (1) was the interaction term *AI*_*p*_ × *mechanisms*_*i*_, which was used to test the potential channels through which AI took an effect. It is worth noting that the direct effect of *AI*_*p*_ was absorbed by the province fixed effects. We employed a variety of estimation methods in empirical analysis and used a stepwise approach when adding control variables. To be specific, after checking the assumptions of ordinary least squares (OLS) [please see Appendix A in the Additional file], we employed the OLS method when the dependent variables were the time to the peak of the cumulative confirmed cases (*TTP*) and the number of local policies on production resumption (*Number of policies*). For limited dependent variables, we used the Tobit model for the continuous dependent variable censored at the value of 0, case fatality rate (*CFR*); the Probit model for the dummy variable, whether there were severe cases (*Severe cases*); and the Poisson model for the non-negative dependent variable, the time span to having such policies (*Time span*).

## Results

### Descriptive analysis

Table 2 showed the summary statistics for all the variables. As for the dependent variables, we could see that the time to the peak of the cumulative confirmed cases had a relatively small coefficient of variation (CV≈0.534). Specifically, almost 50 percent of cities spent less than 20 days controlling the spread of the epidemic. Also, more than 50 percent of cities had no deaths or severe cases. These findings suggested that the epidemic prevention and control practices during the first wave of COVID-19 in mainland China were effective. When it comes to the work resumption, we noted that a large number of policies were introduced as guidelines for reopening, with a maximum of 171 and a median of 36. As for the time span to having the first such policy, we observed the 75^th^ quantile of 9. Besides, the results also showed that there was a large variation in the level of AI development across provinces, with a minimum of 3.091 and a maximum of 9.919 in Table 2. We also found that more than 80 percent of AI patents were concentrated in 10 provinces (including Guangdong, Beijing, Jiangsu, Shanghai, Zhejiang, Shandong, Sichuan, Anhui, Hubei, and Shaanxi). As for the control variables, we observed that the variables varied greatly by region. For example, 28.9 percent of cities were locked down because of COVID-19 during the sample period.

**Table 2:** Descriptive statistics

Variable	Obs	Mean	Std. Dev.	Min	p25	p50	p75	Max
*Dependent variables*								
TTP	304	18.950	10.120	0	12	21	26	43
CFR (%)	304	0.859	2.250	0.000	0.000	0.000	0.000	16.670
Severe cases	304	0.339	0.474	0	0	0	1	1
Number of policies	304	38.730	16.290	14	29	36	45	171
Time span	304	6.595	3.618	0	4	7	9	20
*Independent variable*								
AI	31	7.129	1.434	3.091	6.291	7.197	7.916	9.919
*Mechanism variables*								
Migration	304	1.949	8.342	0.000	0.000	0.000	0.926	89.330
Public transit volume	285	9.080	1.302	4.043	8.390	9.000	9.677	12.720
Number of COVID-19 hospitals	292	0.308	0.435	0.000	0.122	0.200	0.328	5.107
Number of firms	290	5.509	1.286	1.386	4.745	5.509	6.265	9.002
*Macro characteristics*								
GDP per capita	287	11.030	0.583	9.792	10.640	11.010	11.400	15.680
Population density	292	0.078	0.071	0.000	0.032	0.062	0.102	0.565
*Health care resources*								
Proportion of public employment	286	187.400	91.080	43.159	126.500	167.700	221.800	663.667
Number of Three-A hospitals	297	0.009	0.013	0.000	0.000	0.004	0.010	0.074
*Epidemic severity*								
Lockdown	304	0.289	0.454	0	0	0	1	1
Confirmed cases	304	3.099	1.596	0.000	2.197	3.135	3.932	8.166

Table 3 presented the Spearman correlation coefficients among the main variables. We also checked the Pearson correlation coefficients among independent variables [see Appendix B in the Additional file]. In addition, we examined the variance inflation factor (VIF) of each independent variable in our regressions [see the bottom row of Tables 4, 5 and 6]. The results of these tests showed that multicollinearity was not a serious concern in our regressions.

**Table 3:** The Spearman correlation among the main variables

	TTP	CFR (%)	Severe cases	Number of policies	Time span
Migration	0.660^a^	0.317^a^	0.128^b^	0.251^a^	-0.280^a^
Public transit volume	0.435^a^	0.149^b^	0.257^a^	0.224^a^	-0.079
Number of COVID-19 hospitals	-0.067	0.027	-0.211^a^	0.087	0.119^c^
Number of firms	0.488^a^	0.135^b^	0.198^a^	0.253^a^	-0.332^a^
GDP per capita	0.322^a^	0.108^c^	0.165^a^	0.198^a^	-0.248^a^
Population density	0.386^a^	0.139^b^	0.155^b^	0.121^b^	-0.116^c^
Proportion of public employment	-0.178^a^	0.044	0.061	-0.033	0.177^a^
Number of Three-A hospitals	0.319^a^	0.218^a^	0.157^a^	0.092	-0.015
Lockdown	0.239^a^	0.188^a^	-0.002	0.026	-0.087
Confirmed cases	0.813^a^	0.405^a^	0.187^a^	0.319^a^	-0.326^a^

^a^, ^b^ and ^c^ indicate statistical significance at the 1%, 5% and 10% levels, respectively

**Table 4:** The effect of AI on the screening and detection of COVID-19

*Dependent variable*	TTP							
	Cross-border mobility				Within-city mobility			
	(1)	(2)	(3)	(4)	(5)	(6)	(7)	(8)
*Interaction term*								
AI × Migration	-0.481	-0.755^b^	-0.693^c^	-0.748^b^				
	(0.444)	(0.037)	(0.062)	(0.019)				
AI × Public transit volume					0.344*	0.226	0.285	-0.314
					(0.094)	(0.347)	(0.265)	(0.112)
*Mechanism variables*								
Migration	3.882	5.979^b^	5.508^c^	5.709^b^		0.154^b^	0.158^b^	-0.072
	(0.423)	(0.033)	(0.055)	(0.020)		(0.032)	(0.038)	(0.149)
Public transit volume		3.097^a^	3.157^a^	0.536	0.754	1.447	0.963	2.613^c^
		(0.000)	(0.000)	(0.201)	(0.611)	(0.410)	(0.617)	(0.066)
Number of COVID-19 hospitals		-0.041	0.252	0.571		-0.007	0.451	0.625
		(0.964)	(0.792)	(0.424)		(0.994)	(0.646)	(0.406)
*Macro characteristics*								
GDP per capita		-0.023	0.256	1.427^b^		-0.198	0.120	1.475^a^
		(0.984)	(0.819)	(0.010)		(0.868)	(0.916)	(0.008)
Population density		12.232^b^	11.532^c^	2.019		11.939^b^	11.090^c^	1.346
		(0.039)	(0.053)	(0.643)		(0.045)	(0.062)	(0.756)
*Health care resources*								
Proportion of public employment			-0.007	-0.003			-0.010^c^	-0.003
			(0.198)	(0.515)			(0.074)	(0.467)
Number of Three-A hospitals			-17.246	-85.166^c^			2.850	-78.560^c^
			(0.783)	(0.065)			(0.963)	(0.093)
*Epidemic severity*								
Lockdown				0.037				-0.020
				(0.965)				(0.982)
Confirmed cases				5.956^a^				6.098^a^
				(0.000)				(0.000)
Province fixed effects	Yes	Yes	Yes	Yes	Yes	Yes	Yes	Yes
Observations	300	273	270	270	279	273	270	270
*R*-squared	0.372	0.528	0.521	0.749	0.520	0.523	0.519	0.746
Mean VIF	4.19	2.53	2.83	2.90	1.03	1.21	1.44	1.60

^a^, ^b^ and ^c^ indicate statistical significance at the 1%, 5% and 10% levels, respectively. Robust *p*-values were reported in parentheses. When using the Benjamini–Hochberg method, we set the FDR level as 0.05 and the adaptive rejection threshold for Column (4) was calculated to be 0.02

### Regression results

Tables 4, 5 and 6 reported the results of the multivariate analysis. To explore the potential mechanisms underlying the effect of AI on epidemic prevention and control, we focused on the role of AI in the screening and detection of COVID-19 in Table 4, the diagnosis and treatment of COVID-19 in Table 5, and the monitoring and evaluation of the epidemic in Table 6, respectively. In all tables, we followed a stepwise procedure to add the control variables. For each dependent variable, we first included only the province fixed effects, then added variables on local macro characteristics, health care resources, and epidemic severity step by step in the following columns.

**Table 5:** The effect of AI on the diagnosis and treatment of COVID-19

*Dependent variable*	CFR (%)				Severe cases			
	(1)	(2)	(3)	(4)	(5)	(6)	(7)	(8)
*Interaction term*								
AI × Number of COVID-19 hospitals	-1.520	-1.374	-1.324	-1.116	-0.398	-0.161	-0.061	-0.096
	(0.201)	(0.257)	(0.258)	(0.406)	(0.217)	(0.626)	(0.855)	(0.809)
*Independent variable*								
AI	0.897^b^	0.347	0.532	-0.029	0.145^c^	0.031	0.017	-0.056
	(0.034)	(0.485)	(0.301)	(0.962)	(0.093)	(0.751)	(0.865)	(0.624)
*Mechanism variables*								
Number of COVID-19 hospitals	10.155	8.916	8.396	7.028	1.339	-0.181	-1.084	-1.039
	(0.246)	(0.316)	(0.331)	(0.479)	(0.558)	(0.940)	(0.658)	(0.720)
Migration		0.190^a^	0.190^a^	-0.016		-0.109^b^	-0.102^b^	-0.280^a^
		(0.000)	(0.000)	(0.668)		(0.020)	(0.014)	(0.009)
Public transit volume		0.908^c^	0.717	-0.420		0.265^a^	0.357^a^	0.246^b^
		(0.054)	(0.260)	(0.542)		(0.001)	(0.001)	(0.034)
*Macro characteristics*								
GDP per capita		-0.263	-0.581	-0.723		0.124	0.101	0.166
		(0.815)	(0.651)	(0.604)		(0.404)	(0.525)	(0.296)
Population density		9.855^c^	10.484^c^	6.440		0.161	0.357	-0.328
		(0.077)	(0.064)	(0.227)		(0.902)	(0.787)	(0.822)
*Health care resources*								
Proportion of public employment			0.007	0.011^c^			0.001	0.002^c^
			(0.292)	(0.063)			(0.247)	(0.079)
Number of Three-A hospitals			22.732	23.250			-12.384	-9.619
			(0.650)	(0.621)			(0.192)	(0.300)
*Epidemic severity*								
Lockdown				0.329				-0.317
				(0.844)				(0.141)
Confirmed cases				2.474^a^				0.412^a^
				(0.000)				(0.000)
Observations	292	278	275	275	292	278	275	275
Pseudo *R*-squared	0.005	0.045	0.046	0.077	0.056	0.121	0.129	0.177
Mean VIF	1.01	1.15	1.40	1.59	1.01	1.15	1.40	1.59

^a^,^b^ and ^c^ indicate statistical significance at the 1%, 5% and 10% levels, respectively. Robust *p*-values were reported in parentheses. We didn’t include the province fixed effects in Table 5 due to the incidental parameters problem when using nonlinear models

**Table 6:** The effect of AI on the monitoring and evaluation of COVID-19

*Dependent variable*	Number of policies				Time span							
	(1)	(2)	(3)	(4)	(5)				(6)	(7)		(8)
*Interaction term*												
AI × Number of firms	1.831^a^	2.052^a^	1.792^a^	1.692^a^	-0.013^b^				-0.014^b^	-0.011^b^		-0.011^b^
	(0.003)	(0.001)	(0.003)	(0.003)	(0.034)				(0.019)	(0.044)		(0.037)
*Mechanism variables*												
Number of firms	-9.187^b^	-13.474^a^	-12.141^a^	-11.592^a^	0.069^b^			0.103^b^		0.089^b^	0.089^b^	
	(0.013)	(0.001)	(0.003)	(0.003)	(0.048)			(0.014)		(0.023)	(0.022)	
Migration		0.140^b^	0.090 ^a^	0.058^b^				-0.001^c^		-0.001^c^	-0.001^c^	
		(0.032)	(0.008)	(0.035)				(0.097)		(0.066)	(0.057)	
Public transit volume		2.483 ^a^	1.296 ^a^	0.987^b^				-0.022^b^		-0.013^b^	-0.013^b^	
		(0.001)	(0.010)	(0.026)				(0.018)		(0.039)	(0.042)	
Number of COVID-19 hospitals		-0.223	-0.697	-0.601				0.002		0.006	0.005	
		(0.809)	(0.360)	(0.424)				(0.823)		(0.269)	(0.340)	
*Macro characteristics*												
GDP per capita		0.155	0.072	0.236				0.004		0.000	0.000	
		(0.881)	(0.834)	(0.546)				(0.690)		(0.927)	(0.871)	
Population density		8.332	-1.024	-2.253				-0.124		-0.011	-0.011	
		(0.451)	(0.798)	(0.574)				(0.290)		(0.754)	(0.741)	
*Health care resources*												
Proportion of public employment			0.009^c^	0.009 ^c^						-0.000	-0.000	
			(0.076)	(0.073)						(0.407)	(0.427)	
Number of Three-A hospitals			105.786	97.301						-0.660	-0.660	
			(0.116)	(0.145)						(0.515)	(0.530)	
*Epidemic severity*												
Lockdown				0.864							-0.005	
				(0.335)							(0.557)	
Confirmed cases				0.719^b^							0.001	
				(0.018)							(0.804)	
Province fixed effects	Yes	Yes	Yes	Yes			Yes	Yes		Yes	Yes	
Observations	286	271	267	267			264	250		246	246	
*R*-squared	0.741	0.753	0.876	0.878			0.976	0.977		0.991	0.991	
Pseudo *R*-squared							0.249	0.253		0.257	0.257	
Mean VIF	1.00	1.39	1.65	1.80			1.00	1.39		1.65	1.80	

^a^, ^b^ and ^c^ indicate statistical significance at the 1%, 5% and 10% levels, respectively. Robust *p*-values were reported in parentheses. In Column (4), we set the FDR level as 0.05 and the adaptive rejection threshold was calculated to be 0.009. In Column (8), the *p*-value of the AI interaction was smaller than the calculated threshold 0.073 when setting the FDR level as 0.2

Table 4 showed the estimates of the role of AI in the screening and detection of COVID-19. The results in Columns (1) to (4) of Table 4 showed that the effect of AI on the time to the peak of the cumulative confirmed cases (*TTP*) was significantly larger in cities with higher cross-border mobility. In particular, in Column (4) with all controls, the coefficient of the interaction term of interest was -0.748 with a *p*-value of 0.019. To reduce the multiplicity problems, we adjusted significance levels for multiple tests using the Benjamini–Hochberg method based on a desired false discovery rate (FDR), which could balance the cost and benefit of reducing the type I error more effectively than traditional multiple testing methods such as Bonferroni correction [45, 46]. The results in Column (4) were robust to this adjustment, given a FDR level of 0.05 and a calculated threshold of 0.02. Given the mean value of *Migration* (1.949) and the estimated coefficient of the interaction term (-0.748), we estimated that cities with the average intensity of migration rate on average had 1.5 (0.748 × 1.949≈1.458) fewer days of TTP when the number of AI patents doubled, holding other factors constant. By contrast, in the rest of the columns in Table 4, the results showed that AI had a small effect on the time to the peak of the cumulative confirmed cases in cities with high within-city mobility. Results in Table 4 demonstrated that AI can play an important role in screening and detecting the disease, especially in cities with higher cross-border mobility.

As for the control variables, the results showed that cross-border mobility (*Migration*), within-city mobility (*Public transit volume*), population density (*Population density*), and the number of confirmed cases (*Confirmed cases*) had positive effects on *TTP* across columns. Also, *TTP* was sensitive to economic conditions (*GDP per capita*), in line with the effect of economic activities on the transmission of viral diseases documented in Adda (2016) [30] and Qiu et al. (2020) [41]. In terms of health care resources, *TTP* was negatively correlated with more health care resources, including the density of the employment in public sectors (*Proportion of public employment*) and the number of Three-A hospitals per 10,000 people (*Number of Three-A hospitals*), though the coefficients were low significant.

Table 5 examined the role of AI in the diagnosis and treatment of COVID-19. The results in Columns (1) to (4) and Columns (5) to (8) showed the effect of AI on the case fatality rate (*CFR*) and the probability of having severe cases (*Severe cases*), respectively. In Table 5, we found very limited evidence for the effect of AI on the diagnosis and treatment of COVID-19. Across all columns, the coefficients of the interaction terms had low significance. This implied that we failed to observe a larger effect of AI on the fatality rate or the probability of having severe cases in cities with more COVID-19 related health care resources. The results also showed that higher fatality rates and more severe cases were more likely to be observed in cities having higher within-city mobility (*Public transit volume*) and more confirmed cases (*Confirmed cases*). We also found some evidence for the negative effect of the level of economic development (*GDP per capita*) on the probability of having severe cases. As for the marginal effects of AI, please see Appendix C in the Additional file.

Table 6 explored the role of AI in the monitoring and evaluation of the epidemic. The results in Table 6 showed that AI made a great difference in the monitoring and evaluation of the epidemic. We found that in cities with a larger number of firms and thus a higher demand or risk for reopening, a higher level of AI development was linked to more production resumption policies (Columns 1 to 4 in Table 6) and a shorter time span of having such policies (Columns 5 to 8 in Table 6). In particular, given the mean value of *Number of firms* (5.509) and the estimated coefficients of the interaction term of interest in Column (4) (1.692), we estimated that cities with the average number of industrial enterprises on average had 9 (1.692 × 5.509≈9.321) more policies for production resumption when the number of AI patents doubled. Also, as shown in Column (8), the time span of introducing the first work resumption policy shortened by about 1.5 h (0.011 × 5.509≈0.061) when the number of AI patents doubled in cities with the average number of industrial enterprises, holding other factors constant. We also found that more production resumption policies and shorter time spans were correlated with a smaller number of firms, higher cross-border and within-city mobility, and more health care resources. In addition, our main findings were robust to using the Benjamini–Hochberg method when setting the FDR level as 0.05 and 0.2 for Column (4) and Column (8), respectively.

We conducted a battery of robustness checks to ensure the validity of our results. We checked whether our results were robust to alternative samples, for example, excluding all cities in Hubei province (where Wuhan was located). We also used an alternative measure of the local AI development, which was the logarithm of the cumulative number of pre-grant publications of AI-related patents from 2012 to 2019 at the provincial level. The results were qualitatively similar to those presented in the paper. Please see Appendix D in the Additional file for details.

## Discussion

This study provided the systematic empirical evidence for the effect of AI on the prevention and control of COVID-19. Using data on the first wave of COVID-19 in China, we found that local AI development was important not only for the screening and detection of COVID-19, but also for the monitoring and evaluation of the epidemic evolution. Our results could lend strong support to the proposition of the applications of AI against the epidemic in some of the previous reports [14, 47, 48] and descriptive studies [19, 20, 49, 50].

Our results in Columns (1) to (4) of Table 4 showed that AI played an important role in the fight against COVID-19 by effectively screening and detecting the disease in cities with high cross-city mobility. In the first wave of COVID-19 in China, most cases have been shown to be related to Wuhan [20, 38, 39, 51]. In this situation, high-tech methods based on AI were especially important for cross-border contact tracing and case isolation [52, 53]. However, according to the coefficients of the interaction terms in Columns (5) to (8) of Table 4, we found a small effect of AI on the screening and detection of COVID-19 in cities with high within-city mobility. There might be two reasons for this result. First, in response to the epidemic outbreak, most Chinese cities raised the public health emergency level to the highest within a short period (23 January 2020 to 26 January 2020). Since then, intensive measures have been implemented within each city, including travel restrictions [43], encouraging people to stay at home instead of mass gathering [54], the closure of gathering places like schools [55], and even locking down communities [56]. These methods effectively limited the within-city population mobility. Second, due to the openness and transparency of the epidemic data, people quickly realized the seriousness of the epidemic and took actions to reduce unnecessary outings [57]. Therefore, AI made little difference even in cities with high within-city mobility in the recent past year.

In Table 5, we found limited empirical evidence for the effect of AI on the diagnosis and treatment of COVID-19, though there has been wide recognition of AI techniques for lung imaging features. One possible reason might be that we used the case fatality rate to measure the effect of AI in COVID-19 treatment. The case fatality rate of COVID-19 in China was not high. In the first wave of COVID-19, almost all medical resources in China were allocated to the treatment of critically ill patients, which increased the healthcare resource accessibility and lowered the case fatality rate [42]. Also, with timely detection and treatment, most confirmed cases had not turned into severe. In fact, according to Table 2, there was no death case or severe case in more than 50 percent of cities.

Our results on the importance of AI in making policies for production resumption, illustrated in Table 6, suggested that policy decisions should be guided by science rather than optimistic guesses [33]. Spatial epidemiology, using spatial data and methods, has been used to describe and analyze geographical variations in diseases and their risk factors [58, 59]. We showed that incorporating digital, AI and real-time spatiotemporal data into traditional spatial epidemiological research, like epidemic maps, could give local governments a more solid foundation to make policies related to work and production resumption. In addition, the applications of robots to disinfect, patrol, and transport can be very useful for ensuring the effectiveness of COVID-19 prevention and control after the resumption of work. Therefore, AI applications in public health might not only be a healthcare innovation developed in response to local epidemic emergency, but also the key to achieve the optimal way of balancing the public health and economic benefits of health interventions.

Our findings provided several important policy implications for the ongoing epidemic prevention and control. First, AI should be used to improve the speed of the public emergency response of local governments. In China, though the Information System for Disease Control and Prevention has been built since 2003, it did not work well in this pandemic due to the system design which simply copied the original manual epidemic report process [60]. Studies have shown that the epidemic size would be one-third now if public health interventions could have been carried out 5 days earlier [61]. Therefore, updating the system with AI and making the use of intelligent reporting can be a promising way to have effective identification and early warnings.

Second, more AI applications should be used to screen and detect the disease. Though COVID-19 vaccines are becoming more available, the efficacy of COVID-19 vaccines highly depends on various factors, ranging from vaccine potency, implementation, to virus mutations [6, 62, 63]. It will take a long time to return to its pre-epidemic normalcy after mass inoculations [6]. Furthermore, vaccine-efficacy studies tend to focus on the ability to prevent severe disease and death [64]. They cannot lessen the spread of infection or decrease the chances of viral mutation. These insights combined emphasize the fact that timely screening and early detection are still of pivotal importance in efforts against COVID-19. As a result, we should further promote the applications of AI for long-term epidemic prevention and control, such as more effective detection of the asymptomatic based on interaction-based continuous learning and inference of individual probability (CLIIP) [65]. Meanwhile, better products (or ways) for screening and detecting with AI techniques are also required to prepare against these new variants.

Third, the local government may consider using more AI techniques to improve the capability of decision-making and emergency management. The time to relax COVID-19 related restrictions is vital to reduce the rebound risk. It has been argued that the main reason why Chile, among the top five in the world for vaccination rates, experienced viral rebound was a wrong time to relax contact restrictions. Therefore, government officials have a responsibility to accurately assess the current status of the epidemic and help people understand the imperative to continue their preventive health measures. Our results showed that AI could play an important role in this matter. For example, AI technology can quickly land in scenarios with data accumulation, like creating an epidemic map to monitor the development of an epidemic. In addition, AI may help to improve the timeliness and comprehensiveness of government information disclosure. Also, in the first wave of the epidemic in China, there were material deployment difficulties and rapid price increases. More government policies should be implemented to promote the use of unmanned aerial vehicles, industrial robots, and service robots to deal with future challenges.

Last but not least, though we failed to provide evidence for the effect of AI on the diagnosis and treatment of COVID-19, we believe that more actions should be taken to accelerate the integration of AI into the medical industry. As there has been a long-term unbalance between the supply and demand of health care resources in China and other developing countries like India [42, 62, 66], the implementation of AI applications can be useful to improve the efficiency of the health care system by for example employing intelligent consultation system. Further, more policy efforts could be put into promoting the so-called “AI + medical” framework [67–69] to improve the development of medical technology, diagnosis and treatment capabilities, the R&D of new drugs, and the vaccines in preventing the new COVID-19 strains with higher transmissibility. For example, “Visual Presentation of Pneumonia Lesions with 3D Color Images” is one of the successful cases.

Admittedly, there are several limitations in our study. First, due to data availability, our data was cross-sectional in nature, preventing us from characterizing the dynamic effects of AI in the fight against COVID-19. We are also restricted to showing the correlation rather than a causal relationship between AI and the epidemic prevention and control of COVID-19. Second, because of the problem of underreporting and undertesting of COVID-19 cases [70, 71], the importance of AI in the epidemic prevention and control was probably underestimated in our paper. Third, we focused on three potential mechanisms of AI in the epidemic prevention and control. With the evolution of COVID-19 around the world, the scenarios of AI applications may vary. For instance, the application of AI in community screening has become fewer in China, while the use of AI for medical research like pharmaceutical R&D is increasing. Similarly, the application of AI on community screening and treatment would increase during the second wave of COVID-19 in India. It would be promising to analyze the larger scope of AI applications in the epidemic prevention and control in the future.

## Supplementary Information

**Additional file 1:** **Appendix A.** Checking the assumptions of the OLS regressions. **Appendix B.** Spearman correlation among independent variables. **Appendix C.** Marginal effects in Table 5. **Appendix D.** Further robustness checks. 

## Data Availability

The datasets used in the current study are available from the corresponding author on reasonable request.

## Abbreviations

TTP: Time to the peak of the cumulative confirmed cases
CFR: Case fatality rate
AI: Artificial intelligence
COVID-19: Coronavirus disease 2019
GDP: Gross domestic product
BiLSTM: Bidirectional long short-term memory
DNN: Dynamic neural network
OLS: Ordinary least squares
VIF: Variance inflation factor
FDR: False discovery rate
