# Supplementary material for: Artificial intelligence against the first wave of COVID-19: evidence from China
Source: BMC Health Serv Res. 2022 Jun 10;22:767. doi: 10.1186/s12913-022-08146-4 (PMC9186483; doi:10.1186/s12913-022-08146-4)
Supplement: Supplementary file 1 — Additional file 1: Appendix A. Checking the assumptions of the OLS regressions. Appendix B. Spearman correlation among independent variables. Appendix C. Marginal effects in Table 5. Appendix D. Further robustness checks. [file 12913_2022_8146_MOESM1_ESM.docx]

Supplementary Materials

## Appendix A: Checking the assumptions of the OLS regressions

This part tested the assumptions of the OLS regressions, including linearity, zero conditional mean error, homoscedasticity, and non-multicollinearity. We conducted the tests for regressions where the OLS estimation method was employed and all control variables were included.

For the assumption of linearity, the top panel in Fig. A presented the results by plotting the *observed predicted values*. It was shown that scatter points symmetrically distributed along the 45-degree lines (the red lines), suggesting that the linearity assumption held.

To test the assumption of zero conditional mean error, we drew the scatter plots of the residuals against the predicted values. The bottom panel in Fig. A presented the results. We found that scatter points randomly distributed around zero. The results lent us confidence that the assumption of zero conditional mean error held.

As for homoscedasticity, the left bottom panel of Fig. A showed that the assumption of homoscedasticity held for regressions in Table 4. This was further verified by the results in Table A where we performed the the Breusch-Pagan test for heteroscedasticity. When it came to the regression in Column (4) of Table 6, both the scatter plot (the right bottom panel in Fig. A) and the result of the Breusch-Pagan test (the last column in Table A) showed the existence of heteroskedasticity, we thus reported the robust standard errors.

The assumption of the non-existence of multicollinearity was tested by calculating the Variance Inflation Factor (VIF). The results were reported in Table A. In addition, we provided the Pearson correlation coefficients among independent variables in Table B. The results of these tests showed that multicollinearity was not a serious concern in our regressions.

As for the assumptions of random sample and normality, we proposed that they could be met to a large extent in our data. First, our data was cross-sectional in nature. According to Wooldridge (2015), an important feature of cross-sectional data is that we can often assume that they are obtained by random sampling. Second, our sample size was relatively large (almost 300 observations). The central limit theorem (CLT) implied that the assumption of normality could be met even if the errors were not from a normal distribution (Casson and Farmer, 2014). In other words, violations of the normality assumption would not pose a serious concern (Schmidt and Finan, 2018).

In summary, these results showed that the OLS estimation method was appropriate when the dependent variables were the time to the peak of the cumulative confirmed cases (*TTP*) (all columns in Table 4) and the number of policies on production resumption (*Number of policies*) (Columns 1 to 4 in Table 6). As for the limited dependent variables, we have turned to nonlinear models instead of OLS.

**References:**

1. Casson RJ, Farmer LD. **Understanding and checking the assumptions of linear regression: a primer for medical researchers**. *Clinical & experimental ophthalmology* 2014, **42**(6):590-596.
2. Schmidt AF, Finan C. **Linear regression and the normality assumption**. *Journal of clinical epidemiology* 2018, **98**:146-151.
3. Wooldridge JM. **Introductory econometrics: A modern approach**: Cengage learning; 2015.

### Fig. A Tests on linearity, zero conditional mean error, and heteroscedasticity

### Table A Tests on multicollinearity and heteroscedasticity

|  | Column (4) in Table 4 | Column (8) in Table 4 | Column (4) in Table 6 |
| --- | --- | --- | --- |
| Mean VIF | 2.90 | 1.60 | 1.80 |
| Breusch-Pagan test for Heteroscedasticity | 0.808 | 0.700 | 0.000 |

## Appendix B: Spearman correlation among independent variables

### Table B Spearman correlation coefficients among independent variables

|  | AI | Migration | Public transit volume | Number of COVID-19 hospitals | Number of firms | GDP per capita | Population density | Proportion of public employment | Number of Three-A hospitals | Lockdown | Confirmed cases |
| --- | --- | --- | --- | --- | --- | --- | --- | --- | --- | --- | --- |
| AI | 1 |  |  |  |  |  |  |  |  |  |  |
| Migration | 0.186*** | 1 |  |  |  |  |  |  |  |  |  |
| Public transit volume | 0.185*** | 0.439*** | 1 |  |  |  |  |  |  |  |  |
| Number of COVID-19 hospitals | 0.025 | -0.097 | -0.195*** | 1 |  |  |  |  |  |  |  |
| Number of firms | 0.643*** | 0.487*** | 0.570*** | -0.250*** | 1 |  |  |  |  |  |  |
| GDP per capita | 0.297*** | 0.285*** | 0.525*** | -0.133** | 0.563*** | 1 |  |  |  |  |  |
| Population density | 0.334*** | 0.431*** | 0.448*** | -0.166*** | 0.521*** | 0.302*** | 1 |  |  |  |  |
| Proportion of public employment | -0.360*** | -0.101* | -0.050 | 0.163*** | -0.370*** | 0.186*** | -0.220*** | 1 |  |  |  |
| Number of Three-A hospitals | 0.123** | 0.317*** | 0.622*** | -0.308*** | 0.450*** | 0.473*** | 0.351*** | -0.011 | 1 |  |  |
| Lockdown | 0.024 | 0.255*** | 0.281*** | -0.161*** | 0.229*** | 0.256*** | 0.167*** | 0.013 | 0.312*** | 1 |  |
| Confirmed cases | 0.379*** | 0.783*** | 0.472*** | -0.074 | 0.599*** | 0.307*** | 0.468*** | -0.253*** | 0.349*** | 0.267*** | 1 |

Note: ***, ** and * indicate statistical significance at the 1%, 5% and 10% levels, respectively.

## Appendix C: Marginal effects in Table 5

### Table C Marginal effects of AI on the diagnosis and treatment of COVID-19

| *Dependent variable* | CFR (%) | | | |  | Severe cases | | | |
| --- | --- | --- | --- | --- | --- | --- | --- | --- | --- |
|  | (1) | (2) | (3) | (4) |  | (5) | (6) | (7) | (8) |
| *Interaction term* |  |  |  |  |  |  |  |  |  |
| AI× Number of COVID-19 hospitals | -1.520 | -1.374 | -1.324 | -1.116 |  | -0.142 | -0.054 | -0.021 | -0.028 |
|  | (0.201) | (0.257) | (0.258) | (0.406) |  | (0.217) | (0.626) | (0.855) | (0.809) |
| *Independent variable* |  |  |  |  |  |  |  |  |  |
| AI | 0.897** | 0.347 | 0.532 | -0.029 |  | 0.0517* | 0.0103 | 0.006 | -0.016 |
|  | (0.034) | (0.485) | (0.301) | (0.962) |  | (0.093) | (0.751) | (0.865) | (0.624) |
| *Mechanism variables* |  |  |  |  |  |  |  |  |  |
| Number of COVID-19 hospitals | 10.15 | 8.916 | 8.396 | 7.028 |  | 0.478 | -0.061 | -0.366 | -0.298 |
|  | (0.246) | (0.316) | (0.331) | (0.479) |  | (0.558) | (0.940) | (0.657) | (0.720) |
| Migration |  | 0.190*** | 0.190*** | -0.0161 |  |  | -0.037** | -0.034*** | -0.080*** |
|  |  | (0.000) | (0.000) | (0.668) |  |  | (0.011) | (0.008) | (0.000) |
| Public transit volume |  | 0.908* | 0.717 | -0.420 |  |  | 0.089*** | 0.121*** | 0.071** |
|  |  | (0.054) | (0.260) | (0.542) |  |  | (0.001) | (0.001) | (0.036) |
| *Macro characteristics* |  |  |  |  |  |  |  |  |  |
| GDP per capita |  | -0.263 | -0.581 | -0.723 |  |  | 0.042 | 0.034 | 0.048 |
|  |  | (0.815) | (0.651) | (0.604) |  |  | (0.403) | (0.525) | (0.300) |
| Population density |  | 9.855* | 10.480* | 6.440 |  |  | 0.054 | 0.121 | -0.094 |
|  |  | (0.077) | (0.064) | (0.227) |  |  | (0.902) | (0.786) | (0.823) |
| *Health care resources* |  |  |  |  |  |  |  |  |  |
| Proportion of public employment |  |  | 0.007 | 0.011* |  |  |  | 0.000 | 0.001* |
|  |  |  | (0.292) | (0.063) |  |  |  | (0.249) | (0.078) |
| Number of Three-A hospitals |  |  | 22.730 | 23.250 |  |  |  | -4.185 | -2.756 |
|  |  |  | (0.650) | (0.621) |  |  |  | (0.192) | (0.307) |
| *Epidemic severity* |  |  |  |  |  |  |  |  |  |
| lockdown |  |  |  | 0.329 |  |  |  |  | -0.086 |
|  |  |  |  | (0.844) |  |  |  |  | (0.128) |
| Confirmed cases |  |  |  | 2.474*** |  |  |  |  | 0.118*** |
|  |  |  |  | (0.000) |  |  |  |  | (0.000) |
| Observations | 292 | 278 | 275 | 275 |  | 292 | 278 | 275 | 275 |
| Pseudo *R*-squared | 0.005 | 0.045 | 0.046 | 0.077 |  | 0.056 | 0.121 | 0.129 | 0.177 |

Note: ***, ** and * indicate statistical significance at the 1%, 5% and 10% levels, respectively. Robust *P*-values are reported in parentheses.

## Appendix D: Further robustness checks

### D1: Excluding all cities in Hubei province

#### Table D1-4 The effect of AI on the screening and detection of COVID-19

| *Dependent variable* | TTP | | | | | | | | |
| --- | --- | --- | --- | --- | --- | --- | --- | --- | --- |
|  | Cross-border mobility | | | |  | Within-city mobility | | | |
|  | (1) | (2) | (3) | (4) |  | (5) | (6) | (7) | (8) |
| *Interaction term* |  |  |  |  |  |  |  |  |  |
| AI× Migration | -0.094 | -0.554* | -0.496 | -0.913*** |  |  |  |  |  |
|  | (0.830) | (0.094) | (0.139) | (0.001) |  |  |  |  |  |
| AI× Public transit volume |  |  |  |  |  | 0.366* | 0.081 | 0.126 | -0.264 |
|  |  |  |  |  |  | (0.077) | (0.757) | (0.643) | (0.168) |
| *Mechanism variables* |  |  |  |  |  |  |  |  |  |
| Migration | 4.041 | 5.742** | 5.417** | 5.943*** |  |  | 1.538*** | 1.619*** | -0.773*** |
|  | (0.267) | (0.028) | (0.040) | (0.004) |  |  | (0.001) | (0.000) | (0.009) |
| Public transit volume |  | 2.658*** | 2.950*** | 0.658 |  | 0.697 | 2.027 | 1.928 | 2.342* |
|  |  | (0.000) | (0.000) | (0.123) |  | (0.640) | (0.269) | (0.336) | (0.092) |
| Number of COVID-19 hospitals |  | 0.158 | 0.461 | 0.908 |  |  | 0.183 | 0.590 | 0.960 |
|  |  | (0.856) | (0.619) | (0.218) |  |  | (0.835) | (0.536) | (0.223) |
| *Macro characteristics* |  |  |  |  |  |  |  |  |  |
| GDP per capita |  | -0.300 | 0.120 | 1.533*** |  |  | -0.399 | 0.046 | 1.535*** |
|  |  | (0.805) | (0.914) | (0.006) |  |  | (0.748) | (0.968) | (0.006) |
| Population density |  | 13.345* | 13.322* | -0.969 |  |  | 12.998* | 12.929* | -1.613 |
|  |  | (0.062) | (0.068) | (0.858) |  |  | (0.067) | (0.071) | (0.761) |
| *Health care resources* |  |  |  |  |  |  |  |  |  |
| Proportion of public employment |  |  | -0.008 | -0.004 |  |  |  | -0.010* | -0.005 |
|  |  |  | (0.124) | (0.426) |  |  |  | (0.064) | (0.330) |
| Number of Three-A hospitals |  |  | -60.299 | -81.749* |  |  |  | -47.220 | -73.565 |
|  |  |  | (0.354) | (0.079) |  |  |  | (0.470) | (0.127) |
| *Epidemic severity* |  |  |  |  |  |  |  |  |  |
| lockdown |  |  |  | 0.586 |  |  |  |  | 0.213 |
|  |  |  |  | (0.495) |  |  |  |  | (0.806) |
| Confirmed cases |  |  |  | 6.422*** |  |  |  |  | 6.407*** |
|  |  |  |  | (0.000) |  |  |  |  | (0.000) |
| Province fixed effects | Yes | Yes | Yes | Yes |  | Yes | Yes | Yes | Yes |
| Observations | 289 | 262 | 259 | 259 |  | 268 | 262 | 259 | 259 |
| *R*-squared | 0.389 | 0.476 | 0.471 | 0.725 |  | 0.457 | 0.473 | 0.469 | 0.718 |

Note: ***, ** and * indicate statistical significance at the 1%, 5% and 10% levels, respectively. Robust *P*-values are reported in parentheses.

#### Table D1-5 The effect of AI on the diagnosis and treatment of COVID-19

| *Dependent variable* | CFR (%) | | | |  | Severe cases | | | |
| --- | --- | --- | --- | --- | --- | --- | --- | --- | --- |
|  | (1) | (2) | (3) | (4) |  | (5) | (6) | (7) | (8) |
| *Interaction term* |  |  |  |  |  |  |  |  |  |
| AI× Number of COVID-19 hospitals | -2.854* | -1.897 | -1.868 | -1.561 |  | -0.340 | -0.154 | -0.051 | -0.096 |
|  | (0.058) | (0.196) | (0.192) | (0.322) |  | (0.282) | (0.643) | (0.878) | (0.810) |
| *Independent variable* |  |  |  |  |  |  |  |  |  |
| AI | 1.051** | 0.372 | 0.589 | 0.072 |  | 0.142* | 0.028 | 0.013 | -0.056 |
|  | (0.029) | (0.524) | (0.338) | (0.917) |  | (0.096) | (0.769) | (0.897) | (0.623) |
| *Mechanism variables* |  |  |  |  |  |  |  |  |  |
| Number of COVID-19 hospitals | 17.855* | 11.988 | 11.412 | 9.656 |  | 1.028 | -0.225 | -1.143 | -1.041 |
|  | (0.094) | (0.258) | (0.271) | (0.398) |  | (0.646) | (0.926) | (0.640) | (0.720) |
| Migration |  | 0.863*** | 0.821*** | -0.258 |  |  | -0.093 | -0.079 | -0.279*** |
|  |  | (0.003) | (0.006) | (0.472) |  |  | (0.221) | (0.302) | (0.010) |
| Public transit volume |  | 0.687 | 0.575 | -0.399 |  |  | 0.259*** | 0.350*** | 0.246** |
|  |  | (0.254) | (0.475) | (0.655) |  |  | (0.001) | (0.001) | (0.034) |
| *Macro characteristics* |  |  |  |  |  |  |  |  |  |
| GDP per capita |  | -0.680 | -1.054 | -0.942 |  |  | 0.123 | 0.102 | 0.166 |
|  |  | (0.623) | (0.514) | (0.566) |  |  | (0.405) | (0.522) | (0.296) |
| Population density |  | 13.950* | 14.451* | 10.475 |  |  | 0.105 | 0.285 | -0.329 |
|  |  | (0.095) | (0.087) | (0.211) |  |  | (0.938) | (0.835) | (0.822) |
| *Health care resources* |  |  |  |  |  |  |  |  |  |
| Proportion of public employment |  |  | 0.009 | 0.013* |  |  |  | 0.001 | 0.002* |
|  |  |  | (0.278) | (0.066) |  |  |  | (0.256) | (0.079) |
| Number of Three-A hospitals |  |  | 13.896 | 30.870 |  |  |  | -12.606 | -9.622 |
|  |  |  | (0.820) | (0.598) |  |  |  | (0.188) | (0.300) |
| *Epidemic severity* |  |  |  |  |  |  |  |  |  |
| lockdown |  |  |  | 0.234 |  |  |  |  | -0.316 |
|  |  |  |  | (0.901) |  |  |  |  | (0.142) |
| Confirmed cases |  |  |  | 2.792*** |  |  |  |  | 0.412*** |
|  |  |  |  | (0.000) |  |  |  |  | (0.000) |
| Observations | 281 | 267 | 264 | 264 |  | 281 | 267 | 264 | 264 |
| Pseudo *R*-squared | 0.008 | 0.032 | 0.032 | 0.053 |  | 0.052 | 0.0970 | 0.105 | 0.153 |

Note: ***, ** and * indicate statistical significance at the 1%, 5% and 10% levels, respectively. Robust *P*-values are reported in parentheses.

#### Table D1-6 The effect of AI on the monitoring and evaluation of COVID-19

| *Dependent variable* | Number of policies | | | |  | Time span | | | |
| --- | --- | --- | --- | --- | --- | --- | --- | --- | --- |
|  | (1) | (2) | (3) | (4) |  | (5) | (6) | (7) | (8) |
| *Interaction term* |  |  |  |  |  |  |  |  |  |
| AI× Number of firms | 1.841*** | 1.822*** | 1.661*** | 1.639*** |  | -0.013** | -0.012** | -0.011** | -0.011** |
|  | (0.003) | (0.000) | (0.002) | (0.002) |  | (0.034) | (0.018) | (0.037) | (0.031) |
| *Mechanism variables* |  |  |  |  |  |  |  |  |  |
| Number of firms | -9.193** | -12.501*** | -11.463*** | -11.335*** |  | 0.069** | 0.094** | 0.087** | 0.089** |
|  | (0.013) | (0.000) | (0.001) | (0.001) |  | (0.048) | (0.013) | (0.020) | (0.018) |
| Migration |  | 2.429** | 1.413** | 1.320** |  |  | -0.019 | -0.006 | -0.008 |
|  |  | (0.038) | (0.019) | (0.032) |  |  | (0.139) | (0.246) | (0.140) |
| Public transit volume |  | 1.949*** | 1.130** | 1.057** |  |  | -0.019** | -0.012** | -0.014** |
|  |  | (0.001) | (0.020) | (0.029) |  |  | (0.032) | (0.043) | (0.042) |
| Number of COVID-19 hospitals |  | -0.439 | -0.865 | -0.819 |  |  | 0.005 | 0.007 | 0.007 |
|  |  | (0.613) | (0.257) | (0.294) |  |  | (0.604) | (0.223) | (0.231) |
| *Macro characteristics* |  |  |  |  |  |  |  |  |  |
| GDP per capita |  | 0.074 | 0.100 | 0.137 |  |  | 0.004 | -0.000 | 0.001 |
|  |  | (0.945) | (0.784) | (0.721) |  |  | (0.690) | (0.984) | (0.783) |
| Population density |  | 11.671 | -1.323 | -1.799 |  |  | -0.156 | -0.016 | -0.024 |
|  |  | (0.419) | (0.807) | (0.744) |  |  | (0.267) | (0.718) | (0.614) |
| *Health care resources* |  |  |  |  |  |  |  |  |  |
| Proportion of public employment |  |  | 0.010* | 0.010* |  |  |  | -0.000 | -0.000 |
|  |  |  | (0.064) | (0.065) |  |  |  | (0.421) | (0.443) |
| Number of Three-A hospitals |  |  | 79.626 | 79.067 |  |  |  | -0.570 | -0.584 |
|  |  |  | (0.240) | (0.245) |  |  |  | (0.608) | (0.597) |
| *Epidemic severity* |  |  |  |  |  |  |  |  |  |
| lockdown |  |  |  | 0.368 |  |  |  |  | -0.000 |
|  |  |  |  | (0.680) |  |  |  |  | (0.954) |
| Confirmed cases |  |  |  | 0.178 |  |  |  |  | 0.005 |
|  |  |  |  | (0.506) |  |  |  |  | (0.237) |
| Province fixed effects | Yes | Yes | Yes | Yes |  | Yes | Yes | Yes | Yes |
| Observations | 275 | 260 | 256 | 256 |  | 253 | 239 | 235 | 235 |
| R-squared | 0.742 | 0.772 | 0.885 | 0.885 |  |  |  |  |  |
| Pseudo *R*-squared |  |  |  |  |  | 0.252 | 0.256 | 0.260 | 0.260 |

Note: ***, ** and * indicate statistical significance at the 1%, 5% and 10% levels, respectively. Robust *P*-values are reported in parentheses.

### D2: Alternative measure of the local AI development

#### Table D2-4 The effect of AI on the screening and detection of COVID-19

| *Dependent variable* | TTP | | | | | | | | |
| --- | --- | --- | --- | --- | --- | --- | --- | --- | --- |
|  | Cross-border mobility | | | |  | Within-city mobility | | | |
|  | (1) | (2) | (3) | (4) |  | (5) | (6) | (7) | (8) |
| *Interaction term* |  |  |  |  |  |  |  |  |  |
| AI× Migration | -0.473 | -0.762** | -0.699* | -0.771** |  |  |  |  |  |
|  | (0.457) | (0.037) | (0.063) | (0.019) |  |  |  |  |  |
| AI× Public transit volume |  |  |  |  |  | 0.345* | 0.227 | 0.286 | -0.313 |
|  |  |  |  |  |  | (0.094) | (0.350) | (0.267) | (0.116) |
| *Mechanism variables* |  |  |  |  |  |  |  |  |  |
| Migration | 3.737 | 5.908** | 5.438* | 5.753** |  |  | 0.154** | 0.158** | -0.071 |
|  | (0.435) | (0.033) | (0.056) | (0.020) |  |  | (0.032) | (0.038) | (0.150) |
| Public transit volume |  | 3.096*** | 3.155*** | 0.534 |  | 0.799 | 1.481 | 1.001 | 2.555* |
|  |  | (0.000) | (0.000) | (0.202) |  | (0.584) | (0.393) | (0.597) | (0.068) |
| Number of COVID-19 hospitals |  | -0.041 | 0.252 | 0.571 |  |  | -0.008 | 0.449 | 0.627 |
|  |  | (0.964) | (0.792) | (0.423) |  |  | (0.993) | (0.648) | (0.405) |
| *Macro characteristics* |  |  |  |  |  |  |  |  |  |
| GDP per capita |  | -0.022 | 0.258 | 1.431** |  |  | -0.198 | 0.119 | 1.475*** |
|  |  | (0.985) | (0.817) | (0.010) |  |  | (0.868) | (0.917) | (0.008) |
| Population density |  | 12.238** | 11.526* | 2.011 |  |  | 11.932** | 11.084* | 1.359 |
|  |  | (0.039) | (0.053) | (0.644) |  |  | (0.045) | (0.062) | (0.753) |
| *Health care resources* |  |  |  |  |  |  |  |  |  |
| Proportion of public employment |  |  | -0.007 | -0.003 |  |  |  | -0.010* | -0.003 |
|  |  |  | (0.197) | (0.516) |  |  |  | (0.074) | (0.464) |
| Number of Three-A hospitals |  |  | -17.216 | -85.542* |  |  |  | 2.993 | -78.622* |
|  |  |  | (0.783) | (0.064) |  |  |  | (0.962) | (0.092) |
| *Epidemic severity* |  |  |  |  |  |  |  |  |  |
| lockdown |  |  |  | 0.060 |  |  |  |  | -0.022 |
|  |  |  |  | (0.944) |  |  |  |  | (0.980) |
| Confirmed cases |  |  |  | 5.958*** |  |  |  |  | 6.095*** |
|  |  |  |  | (0.000) |  |  |  |  | (0.000) |
| Province fixed effects | Yes | Yes | Yes | Yes |  | Yes | Yes | Yes | Yes |
| Observations | 300 | 273 | 270 | 270 |  | 279 | 273 | 270 | 270 |
| *R*-squared | 0.372 | 0.528 | 0.521 | 0.749 |  | 0.520 | 0.523 | 0.519 | 0.746 |

Note: ***, ** and * indicate statistical significance at the 1%, 5% and 10% levels, respectively. Robust *P*-values are reported in parentheses.

#### Table D2-5 The effect of AI on the diagnosis and treatment of COVID-19

| *Dependent variable* | CFR (%) | | | |  | Severe cases | | | |
| --- | --- | --- | --- | --- | --- | --- | --- | --- | --- |
|  | (1) | (2) | (3) | (4) |  | (5) | (6) | (7) | (8) |
| *Interaction term* |  |  |  |  |  |  |  |  |  |
| AI× Number of COVID-19 hospitals | -1.499 | -1.356 | -1.315 | -1.108 |  | -0.407 | -0.174 | -0.078 | -0.115 |
|  | (0.203) | (0.256) | (0.255) | (0.402) |  | (0.198) | (0.592) | (0.814) | (0.769) |
| *Independent variable* |  |  |  |  |  |  |  |  |  |
| AI | 0.900** | 0.347 | 0.539 | -0.027 |  | 0.143* | 0.028 | 0.014 | -0.060 |
|  | (0.034) | (0.485) | (0.297) | (0.964) |  | (0.094) | (0.772) | (0.887) | (0.600) |
| *Mechanism variables* |  |  |  |  |  |  |  |  |  |
| Number of COVID-19 hospitals | 9.749 | 8.557 | 8.110 | 6.783 |  | 1.338 | -0.118 | -0.979 | -0.929 |
|  | (0.250) | (0.318) | (0.330) | (0.478) |  | (0.540) | (0.959) | (0.680) | (0.739) |
| Migration |  | 0.190*** | 0.190*** | -0.016 |  |  | -0.109** | -0.101** | -0.280*** |
|  |  | (0.000) | (0.000) | (0.668) |  |  | (0.020) | (0.014) | (0.009) |
| Public transit volume |  | 0.907* | 0.714 | -0.423 |  |  | 0.265*** | 0.358*** | 0.246** |
|  |  | (0.054) | (0.261) | (0.540) |  |  | (0.001) | (0.001) | (0.034) |
| *Macro characteristics* |  |  |  |  |  |  |  |  |  |
| GDP per capita |  | -0.262 | -0.583 | -0.726 |  |  | 0.127 | 0.107 | 0.173 |
|  |  | (0.816) | (0.651) | (0.603) |  |  | (0.390) | (0.503) | (0.278) |
| Population density |  | 9.850* | 10.474* | 6.450 |  |  | 0.176 | 0.363 | -0.326 |
|  |  | (0.077) | (0.064) | (0.227) |  |  | (0.893) | (0.783) | (0.823) |
| *Health care resources* |  |  |  |  |  |  |  |  |  |
| Proportion of public employment |  |  | 0.007 | 0.011* |  |  |  | 0.001 | 0.002* |
|  |  |  | (0.290) | (0.064) |  |  |  | (0.264) | (0.086) |
| Number of Three-A hospitals |  |  | 22.863 | 23.439 |  |  |  | -12.511 | -9.728 |
|  |  |  | (0.648) | (0.618) |  |  |  | (0.187) | (0.294) |
| *Epidemic severity* |  |  |  |  |  |  |  |  |  |
| lockdown |  |  |  | 0.327 |  |  |  |  | -0.321 |
|  |  |  |  | (0.845) |  |  |  |  | (0.136) |
| Confirmed cases |  |  |  | 2.473*** |  |  |  |  | 0.414*** |
|  |  |  |  | (0.000) |  |  |  |  | (0.000) |
| Observations | 292 | 278 | 275 | 275 |  | 292 | 278 | 275 | 275 |
| Pseudo *R*-squared | 0.005 | 0.045 | 0.046 | 0.077 |  | 0.056 | 0.122 | 0.129 | 0.178 |

Note: ***, ** and * indicate statistical significance at the 1%, 5% and 10% levels, respectively. Robust *P*-values are reported in parentheses.

#### Table D2-6 The effect of AI on the monitoring and evaluation of COVID-19

| *Dependent variable* | Number of policies | | | |  | Time span | | | |
| --- | --- | --- | --- | --- | --- | --- | --- | --- | --- |
|  | (1) | (2) | (3) | (4) |  | (5) | (6) | (7) | (8) |
| *Interaction term* |  |  |  |  |  |  |  |  |  |
| AI× Number of firms | 1.859*** | 2.090*** | 1.807*** | 1.706*** |  | -0.013** | -0.015** | -0.011** | -0.011** |
|  | (0.003) | (0.001) | (0.003) | (0.003) |  | (0.033) | (0.018) | (0.043) | (0.037) |
| *Mechanism variables* |  |  |  |  |  |  |  |  |  |
| Number of firms | -9.089** | -13.406*** | -11.950*** | -11.407*** |  | 0.068** | 0.103** | 0.088** | 0.087** |
|  | (0.012) | (0.001) | (0.003) | (0.003) |  | (0.045) | (0.013) | (0.023) | (0.022) |
| Migration |  | 0.140** | 0.090*** | 0.058** |  |  | -0.001* | -0.001* | -0.001* |
|  |  | (0.032) | (0.009) | (0.035) |  |  | (0.097) | (0.067) | (0.057) |
| Public transit volume |  | 2.484*** | 1.296** | 0.987** |  |  | -0.022** | -0.013** | -0.013** |
|  |  | (0.001) | (0.010) | (0.027) |  |  | (0.018) | (0.039) | (0.043) |
| Number of COVID-19 hospitals |  | -0.224 | -0.700 | -0.604 |  |  | 0.002 | 0.006 | 0.005 |
|  |  | (0.808) | (0.359) | (0.422) |  |  | (0.817) | (0.265) | (0.336) |
| *Macro characteristics* |  |  |  |  |  |  |  |  |  |
| GDP per capita |  | 0.146 | 0.060 | 0.226 |  |  | 0.004 | 0.000 | 0.000 |
|  |  | (0.887) | (0.861) | (0.563) |  |  | (0.686) | (0.899) | (0.846) |
| Population density |  | 8.317 | -0.995 | -2.226 |  |  | -0.124 | -0.012 | -0.012 |
|  |  | (0.450) | (0.804) | (0.578) |  |  | (0.288) | (0.746) | (0.734) |
| *Health care resources* |  |  |  |  |  |  |  |  |  |
| Proportion of public employment |  |  | 0.009* | 0.009* |  |  |  | -0.000 | -0.000 |
|  |  |  | (0.078) | (0.074) |  |  |  | (0.405) | (0.425) |
| Number of Three-A hospitals |  |  | 105.687 | 97.191 |  |  |  | -0.656 | -0.656 |
|  |  |  | (0.117) | (0.147) |  |  |  | (0.518) | (0.533) |
| *Epidemic severity* |  |  |  |  |  |  |  |  |  |
| lockdown |  |  |  | 0.861 |  |  |  |  | -0.005 |
|  |  |  |  | (0.336) |  |  |  |  | (0.551) |
| Confirmed cases |  |  |  | 0.722** |  |  |  |  | 0.001 |
|  |  |  |  | (0.018) |  |  |  |  | (0.811) |
| Province fixed effects | Yes | Yes | Yes | Yes |  | Yes | Yes | Yes | Yes |
| Observations | 286 | 271 | 267 | 267 |  | 264 | 250 | 246 | 246 |
| R-squared | 0.742 | 0.754 | 0.876 | 0.878 |  |  |  |  |  |
| Pseudo *R*-squared |  |  |  |  |  | 0.249 | 0.253 | 0.257 | 0.257 |

Note: ***, ** and * indicate statistical significance at the 1%, 5% and 10% levels, respectively. Robust *P*-values are reported in parentheses.
